# Robust expression of vault RNAs induced by influenza A virus plays a critical role in suppression of PKR-mediated innate immunity

**DOI:** 10.1093/nar/gkv1078

**Published:** 2015-10-20

**Authors:** Fang Li, Yuhai Chen, Zhaoyuan Zhang, Jing Ouyang, Yi Wang, Ruoxiang Yan, Shile Huang, George Fu Gao, Guijie Guo, Ji-Long Chen

**Affiliations:** 1 CAS Key Laboratory of Pathogenic Microbiology and Immunology, Institute of Microbiology, Chinese Academy of Sciences (CAS), Beijing 100101, China; 2 School of Life Sciences, Anhui University, Hefei 230601, China; 3 College of Animal Sciences, Fujian Agriculture and Forestry University, Fuzhou 350002, China; 4 Department of Biochemistry and Molecular Biology, Louisiana State University Health Sciences Center, Shreveport, LA 71130, USA

## Abstract

Protein kinase R (PKR) is a vital component of host innate immunity against viral infection. However, the mechanism underlying inactivation of PKR by inﬂuenza A virus (IAV) remains elusive. Here, we found that vault RNAs (vtRNAs) were greatly induced in A549 cells and mouse lungs after infection with IAV. The viral NS1 protein was shown to be the inducer triggering the upregulation of vtRNAs. Importantly, silencing vtRNA in A549 cells significantly inhibited IAV replication, whereas overexpression of vtRNAs markedly promoted the viral replication. Furthermore, *in vivo* studies showed that disrupting vtRNA expression in mice significantly decreased IAV replication in infected lungs. The vtRNA knockdown animals exhibited significantly enhanced resistance to IAV infection, as evidenced by attenuated acute lung injury and spleen atrophy and consequently increased survival rates. Interestingly, vtRNAs promoted viral replication through repressing the activation of PKR and the subsequent antiviral interferon response. In addition, increased expression of vtRNAs was required for efficient suppression of PKR by NS1 during IAV infection. Moreover, vtRNAs were also significantly upregulated by infections of several other viruses and involved in the inactivation of PKR signaling by these viruses. These results reveal a novel mechanism by which some viruses circumvent PKR-mediated innate immunity.

## INTRODUCTION

The host mobilizes the innate immune system as the first line of defense against viral infection. The innate immune response involves the recognition of pathogen-associated molecular patterns (PAMPs) by pattern recognition receptors (PRRs) and the activation of cell signaling cascades leading to the production of interferons (IFNs). Protein kinase R (PKR) is an interferon (IFN)-induced and double stranded RNA (dsRNA)-activated Ser/Thr-protein kinase, which plays a key role in antiviral response (1). Once activated, PKR phosphorylates itself and downstream substrates, including the eukaryotic initiation factor 2α-subunit (eIF-2α) and IκB (2,3). Phosphorylated eIF2α causes a dramatic inhibition of viral protein synthesis, thereby blocking viral replication (2,4). In addition, PKR promotes the activation of nuclear factor-kappa B (NF-κB) through phosphorylation of IκB (3). NF-κB, as a transcription factor, positively regulates the transcription of IFN genes and contributes to the expression of IFN-stimulated genes (ISGs) (5,6).

On the other hand, viruses can control or take advantage of the host components to support their own reproduction. For example, various viruses including inﬂuenza virus have evolved multiple strategies to circumvent the activation of antiviral protein PKR (7,8). Influenza A virus (IAV) infection does not activate PKR (9). Instead, IAV represses PKR activity through viral-encoded non-structural protein NS1 and a cellular protein p58^IPK^ (10–12). Previous studies have shown that IAV activates p58^IPK^ by disassociating it from its inhibitor Hsp40, and activated p58^IPK^ inhibits PKR dimerization and phosphorylation through direct interaction between these molecules (13,14). Moreover, the activity of PKR can be remarkably induced by NS1-deleted virus (15). However, the mechanism by which NS1 inhibits PKR activation is still unknown. It has been proposed that NS1 might sequester dsRNA away from PKR (10), but the affinity between NS1 and dsRNA is low (16). Additionally, the requirement of NS1 RNA-binding domain and the direct interaction between NS1 and PKR are controversial according to the reports by different groups of investigators (12,17–19). Therefore, the precise mechanism underlying NS1-mediated inhibition of PKR activity remains to be elucidated.

Recently, growing evidence has implicated a critical role of host noncoding RNAs (ncRNAs) in virus-host interactions (20–25). MicroRNAs, such as miR-122, miR-146a, and miR-155, are crucial for IFN immune response by modulating promoter methylation of suppressor of cytokine signaling 3 (SOCS3), activation of NF-κB and expression of suppressor of cytokine signaling 1 (SOCS1) (26–28). Interestingly, several long noncoding RNAs (lncRNAs) have been shown to possess important functions in antiviral response as well. For example, the stimulus/virus-responsive production of nuclear enriched abundant transcript 1 (NEAT1), lncRNA-CMPK2 and NRAV regulate the expression of genes including those of antiviral factors such as interleukin-8 (IL-8) and several ISGs (22,24,25). However, these identified functional ncRNAs are just the tip of an iceberg when compared with thousands of virus-induced differentially expressed ncRNAs (24).

Vault RNAs (vtRNAs), including vtRNA1-1, vtRNA1-2, vtRNA1-3 and vtRNA2-1 (also known as nc886), belong to noncoding RNAs that are initially characterized as components of large cytoplasmic ribonucleoprotein particles (vaults) with an unusual barrel shaped morphology (29,30). Interest in vaults has been fuelled by their considerable abundance in multidrug-resistant cancer cells and high evolutionary conservation (31). However, previous studies in biological significance of vaults have only focused on the major vault protein (MVP), but not the vtRNAs. Given that most of vtRNAs exist in free form (31,32), and depletion of vtRNAs has no apparent effect on vault particle morphology (33), it is suggested that vtRNAs might function by a non-structural way. Indeed, Persson *et al*. have shown that vtRNA1-1 can be processed to form regulatory small RNAs (svRNAs). One of those svRNAs, svRNAb, employs a miRNA-like mechanism to downregulate CYP3A4, a key enzyme in drug metabolism (34). Recently, it has been reported that expression of vtRNAs is induced by γ-herpesviruses (32,35), implying that vtRNAs might be involved in host-virus interaction. Interestingly, vtRNA2–1 has been shown to be associated with PKR and acts as a novel tumor suppressor in a wide range of cancer cells (36–39). Although it is well known that PKR plays a critical role in host innate immune responses, the involvement of vtRNAs in these processes needs to be determined.

In this study, we investigated the expression of vtRNA using a cDNA microarray analysis of the cellular transcriptional response to IAV and the role of vtRNA during IAV infection. The results showed that IAV induced robust expression of vtRNAs in host through IAV protein NS1. In turn, vtRNAs promoted viral replication by attenuating PKR activity. Apparently, the increased expression of vtRNAs was essential for NS1-mediated inhibition of PKR activation. Furthermore, we found that vtRNAs were also involved in the inactivation of PKR by several other viruses. Our results reveal a novel mechanism of how viruses including IAV overcome PKR-mediated innate immune response through NS1-dependent upregulation of vtRNAs.

## MATERIALS AND METHODS

### Ethics statement

The animal protocol used in this study was approved by the Research Ethics Committee of Institute of Microbiology, Chinese Academy of Sciences (permit number PZIMCAS2012009). All mouse experimental procedures were performed in accordance with the Regulations for the Administration of Affairs Concerning Experimental Animals approved by the State Council of People's Republic of China.

### Viruses and reagents

Influenza virus A/WSN/33 (H1N1), A/PR/8/34 wild type (WT), deltaNS1 (delNS1) and Sendai virus (SeV) were generated and propagated in specific-pathogen-free (SPF) chicken embryo as previously described (40,41). Herpes simplex virus 1 (HSV-1) was propagated in Vero cells as previously described (24). The following antibodies were used in this study: anti-phospho-PKR (Thr446) (Abcam, Cambridge, UK); anti-influenza A virus NP polyclonal antibody was obtained by immunizing rabbits with GST-tagged NP protein. All other antibodies were obtained as previously described (24,42).

### Construction of plasmids and generation of stable cell lines

Eight reverse genetic plasmids for the rescue of influenza virus A/PR/8/34 were kindly provided by Ron A.M. Fouchier (National Influenza Center and Department of Virology, Erasmus MC, Rotterdam, Netherlands). DelNS1 plasmid, a derivative of the PR8 NS segment, was cloned into the vector pHW2000 as previously described (42). The cDNA sequences coding vtRNA1-1, vtRNA1-2, vtRNA1-3 or vtRNA2-1 were subcloned into the BamHI/EcoRI sites of pSIH-H1-GFP lentivirus vector that contains a human H1 RNA polymerase III (Pol III) promoter. Stable cell lines expressing these vtRNAs were generated by using a lentiviral spin infection as previously described (40). The vector expressing NS1 protein of H7N9 (A/chicken/Wuxi/2013) or PR8 was constructed by inserting NS1-ORF into pFlag-CMV5a, and the plasmid expressing NS1 of H5N1 (A/bar-headed goose/Qinghai/2005) was generated by cloning NS1-ORF into pcDNA3.1.

### Antisense oligonucleotides

The antisense oligonucleotides (ASOs) were mixed oligonucleotides containing a backbone phosphorothioate and having 5 or 6 nt (underlined) on each end substituted with 2′-*O*-methyl ribonucleotides (36,43). The sequences of ASOs targeted vtRNAs were as follows: ASO-vtRNA1, 5′-CCGCTGAGCTAAAGCCAGCC-3′; ASO-vtRNA2–1a, 5′-AAAGTCCGGCATGAGGAGGT-3′, and ASO-vtRNA2–1b, 5′-CAGAGATGGACAGATAGAAA-3′; ASO-GFP control, 5′-TCACCTTCACCCTCTCCACT-3′. The sequences of ASOs targeted mvtRNA were as follows: ASO-mvtRNA1, 5′-GGGTTAGGTAAGTGGTTGGTTGTGT-3′, ASO-mvtRNA2, 5′-GCTGGCCCGTCTATCTCTTCCTGGA-3′, ASO-mvtRNA3, 5′-CGGGTTAGGTAAGTGGTTGG-3′. Transfection of ASOs was performed with Lipofectamine™ RNAiMAX reagent (Invitrogen, Carlsbad, CA, USA) per the manufacturer's instructions.

### Cell culture and infection

A549 (human type II alveolar epithelial cells, American Type Culture Collection (ATCC)), 293T (human embryonic kidney cells, ATCC), K562 (human chronic myelogenous leukemia cells, ATCC), MCF7 (human breast adenocarcinoma cells, ATCC), HeLa (human cervical adenocarcinoma cells, ATCC), Huh7 (human hepatocellular carcinoma cells, National Platform of Experimental Cell Resources for Sci-Tech, http://cellresource.cn), NIH/3T3 (mouse embryonic fibroblast cells, ATCC), LLC (mouse Lewis lung carcinoma cells, National Platform of Experimental Cell Resources for Sci-Tech, http://cellresource.cn), 4T1 (mouse mammary tumor cells, ATCC) and MDCK (Madin-Darby canine kidney cells, ATCC) cells were cultured in Dulbecco's Modified Eagle's Medium (DMEM) or RPMI1640 (Gibco-BRL, Inc., Gaithersburg, MD, USA) containing 10% fetal bovine serum (FBS) (Gibco-BRL, Inc., Gaithersburg, MD, USA) supplemented with penicillin (100 U/ml) and streptomycin (100 U/ml) as previously described (24). Cells were infected with influenza A/WSN/33 virus, SeV or HSV-1 at a multiplicity of infection (MOI) of 1–2, unless indicated. After adsorption for 1 h at 37°C, the cells were washed with phosphate-buffered saline (PBS) and cultured in DMEM containing 2 μg/ml trypsin for indicated time.

### Mouse experiments

Female C57BL/6 mice (5–6 weeks old, 18–20 g) were provided by Vital River Laboratory Animal Center (Beijing, China). Mouse experiments were performed as previously described (42). For infection, mice were inoculated intranasally with 5 × 10^4^ plaque-forming units (PFU) of IAV viruses. At the indicated time post infection (p.i.), the mice were then euthanized and their lungs were removed aseptically for further analysis.

### ASO treatment

Female C57BL/6 mice (4–5 weeks old) were inhaled aerosolized chemically modified antisense oligonucleotides (ASOs) targeting mvtRNA or GFP using an air-compressing nebulizer (at an estimated inhalable dose of 3.0 mg/kg). After 24 h, mice were inoculated intranasally with 1 × 10^4^ PFU of the influenza A/WSN/33 virus. At the indicated time post infection, the mice were euthanized and their organs (lung and spleen) were removed aseptically for further analysis.

### HA assay and plaque assay

Hemagglutinin (HA) assay and plaque assay were performed as previously described (40). Brieﬂy, for HA assay, the cell culture supernatants of infected cells were serially diluted with PBS and mixed with an equal volume of 0.5% chicken erythrocytes. The viral titers were counted 20 min later. For plaque assay, MDCK cells were infected with serial dilutions of the supernatants and overlaid with α-minimal essential medium containing 1.5% low melting point agarose and 2 μg/ml TPCK (l-1-tosylamido-2-phenylethyl chloromethyl ketone)-treated trypsin. Plaques were then counted after 72 h incubation at 37°C.

### Cell stimulation, transient transfection and western blotting

For stimulation, cells were transfected with RNA or poly (I:C) using Lipofectamine 2000 (Invitrogen, Carlsbad, CA, USA) for 4 h according to the manufacturer's instructions. For transient transfection, cells were transiently transfected with 2.5 μg plasmids per well of 6-well plate using Vigofect (Vigorous, Beijing, China) according to the manufacturer's instructions. The cells were then cultured for another 30 h for transient expression. Western blotting was performed as previously described (44). Briefly, samples were separated by SDS-polyacrylamide gel electrophoresis, transferred onto a nitrocellulose membrane, and probed with antibodies as indicated.

### RNA preparation, RT-PCR and quantitative real-time PCR (qRT-PCR)

Total RNA was extracted from cells or tissues using TRIzol reagent (Invitrogen, Carlsbad, CA, USA). cDNA was synthesized using 5 μg of total RNA and reverse transcriptase (RT; Promega, Madison, WI, USA), followed by PCR using rTaq DNA polymerase and quantitative PCR using SYBR PremixEx TaqII (TaKaRa, Tokyo, Japan) with the primers shown in Table 1. Primer pairs named mvtRNA-251 and mvtRNA-141 are designed to examine mouse vtRNA. GAPDH was chosen as a reference gene for internal standardization.

**Table 1.** Sequences of primers used in this study

Name	Sequence (5′-3′)
vtRNA1–1 forward	TTAGCTCAGCGGTTACTTCGACAGTTC
vtRNA1–1 reverse	AAAAGGACTGGAGAGCGCCC
vtRNA1–2 forward	GGCTGGCTTTAGCTCAGCGG
vtRNA1–2 reverse	AAAAGAGCTGGAAAGCACCC
vtRNA1–3 forward	ACTTCGCGTGTCATCAAACC
vtRNA1–3 reverse	AAGAGGGCTGGAGAGCGCC
vtRNA2–1 forward	GGGTCGGAGTTAGCTCAAGC
vtRNA2–1 reverse	AAAGGGTCAGTAAGCACCCG
mvtRNA-251 forward	GGACCCGATTGGTCTGTCAT
mvtRNA-251 reverse	GATTCGCAGCGGCAAAAGG
mvtRNA-141 forward	CAGCTTTAGCTCAGCGGTTAC
mvtRNA-141 reverse	AAGGGCCAGGGAGCGCCCGC
NP forward	TCAAACGTGGGATCAATG
NP reverse	GTGCAGACCGTGCTAGAA
NS1 forward	ATTCCTTGATCGGCTTCG
NS1 reverse	GCCTGCCACTTTCTGCTT
IFN-β forward	TGGGAGGCTTGAATACTGCCTCAA
IFN-β reverse	TCCTTGGCCTTCAGGTAATGCAGA
IL29 forward	GGAAGCAGTTGCGATTTAG
IL29 reverse	ATTTGAACCTGCCAATGTG
Mx1 forward	CGTTAGCCGTGGTGATTTAG
Mx1 reverse	CCCTTTCCCAGTACGAAGAC
GAPDH (human) forward	AGAAGGCTGGGGCTCATTTG
GAPDH (human) reverse	AGGGGCCATCCACAGTCTTC
GAPDH (mouse) forward	GCCTCGTCCCGTAGACAAAA
GAPDH (mouse) reverse	CCCTTTTGGCTCCACCCTTC
5S rRNA forward	GGCCATACCACCCTGAACGC
5S rRNA reverse	CAGCACCCGGTATTCCCAGG

### Histopathological analysis

Mouse organs were fixed in 4% paraformaldehyde and embedded in paraffin. Then 4 mm thick sections were prepared and stained with hematoxylin and eosin (HE). The slides were visualized under an Olympus BH-2 microscope (Tokyo, Japan).

### Northern blotting

Total RNA was isolated using Trizol reagent, resolved on a 10% TBE-Urea denaturing gel and electro-transferred to an Immobilon™-Ny+ membrane (Merck Millipore, Germany) at 200 mA for 1 h. After UV-crosslinking, membranes were hybridized with indicated probes at 42°C overnight. All probes were labelled with ^32^P-ATP by T4 polynucleotide kinase (New England Biolabs, Cambridge, MA). Subsequently, the hybridized membranes were washed with 2× SSC/0.5% SDS, exposed to Phosphor-imaging screens (Amersham Biosciences), and scanned by Typhoon FLA7000. 5S rRNA was used as a loading control. The probes used in northern blotting are shown in Table 2.

**Table 2.** Sequences of probes used in analysis of northern blot

Probe	Sequence (5′-3′)
vtRNA1–1 (36)	AAAAGGACTGGAGAGCGCCCG
vtRNA1–2 (32)	AGGTGGTTACAATGTACTCGAAG
vtRNA1–3 (36)	AAGAGGGCTGGAGAGCGCCCG
vtRNA2–1 (36)	AAGGGTCAGTAAGCACCCGCG
5S rRNA (36)	GATCGGGCGCGTTCAGGGTGGTAT

### Statistical analysis

All data represent the mean values ± standard error (mean ± SE). Statistical analysis was performed by Student's *t* test. Differences were considered statistically significant with *P* < 0.05.

## RESULTS

### Robust expression of vtRNAs is induced during IAV infection both *in vitro* and *in vivo*

**Figure 1.**
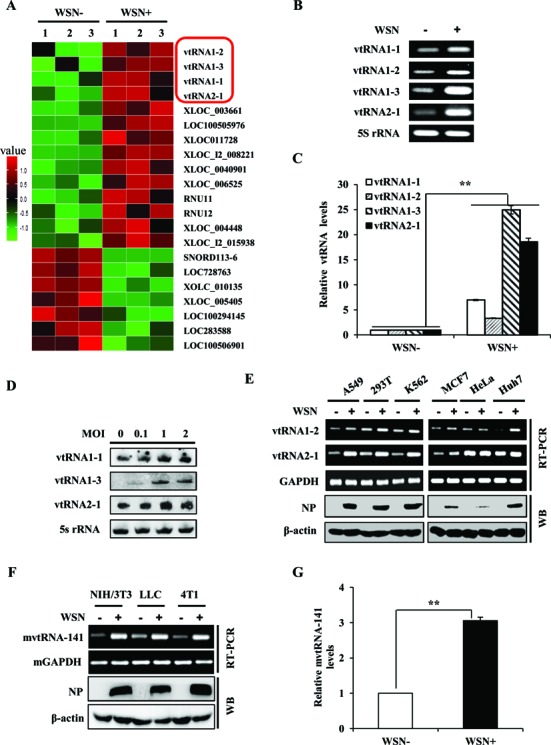
vtRNAs are significantly upregulated during IAV infection both *in vitro* and *in vivo*. (**A**) The differentially expressed ncRNAs in A549 cells infected with or without A/WSN/33 influenza virus were analyzed by a cDNA microarray (http://www.ncbi.nlm.nih.gov/geo/; GEO access number GSE58741). Shown are representative ncRNAs whose expressions were significantly changed. vtRNAs are indicated by red rectangle. (**B**, **C**) A549 cells were infected with or without WSN for 16 h. RT-PCR (B) and qRT-PCR (C) were performed to determine the expression of vtRNAs. (**D**) A549 cells were infected with WSN at indicated MOIs for 16 h. Northern blotting was performed to determine vtRNAs expression. 5S rRNA was used as a loading control. (**E**) The expression of vtRNAs in indicated human cell lines infected with or without WSN for 16 h was examined by RT-PCR. The viral nucleoprotein (NP) was examined by western blotting. (**F**) The mvtRNA expression in mouse cell lines infected with or without WSN for 18 h was examined by RT-PCR. The viral nucleoprotein (NP) was examined by Western blotting. (**G**) C57BL/6 mice intranasally infected with or without WSN (5 × 10^4^ PFU) for 2 days were sacriﬁced, and the lungs were dissected and lysed, followed by qRT-PCR to examine the mvtRNA levels. Shown are representative results from three independent experiments. The error bars represent the SE, **P < 0.01.

To address the relationship between ncRNAs and IAV infection, the differential expression patterns of ncRNAs in human alveolar epithelial cells (A549) infected with or without IAV were analyzed by an annotated cDNA microarray (http://www.ncbi.nlm.nih.gov/geo; GEO access number GSE58741). Interestingly, four vtRNA family members, including vtRNA1-1, vtRNA1-2, vtRNA1-3 and vtRNA2-1, were significantly upregulated upon infection with the influenza A/WSN/33 (H1N1) virus (WSN) (Figure 1A). This was confirmed by RT-PCR and qRT-PCR (Figure 1B, C, Supplementary Figure S1A and B). Furthermore, we observed that vtRNAs were upregulated in a virus dose-dependent manner by analysis of northern blot and qRT-PCR (Figure 1D and Supplementary Figure S1C). Moreover, these vtRNAs were found to be expressed in various human cell lines including A549, 293T, K562, MCF7, HeLa and Huh7 by using RT-PCR, and their expression levels were elevated by IAV infection in all examined cell lines susceptible to the infection (Figure 1E), except for HeLa cell line that is less permissive to IAV replication (45). The increased expression of vtRNA2–1 in IAV-infected host was further confirmed in A549, MCF7 and HEK293 cells by Northern blotting (Supplementary Figure S1D).

In addition, we observed that IAV infection highly induced expression of mouse vtRNA (mvtRNA) in several mouse cell lines (Figure 1F and Supplementary Figure S1E). Similar results were obtained from *in vivo* studies testing lung samples of C57BL/6 mice intranasally infected with WSN by RT-PCR using two primer pairs to detect mvtRNA (Figure 1G, Supplementary Figure S1F, G, H, I and J). These data provide strong evidence that IAV infection causes significant elevation of vtRNAs expression both *in vitro* and *in vivo*.

### Altering expression of vtRNAs has profound effects on IAV replication in A549 cells

**Figure 2.**
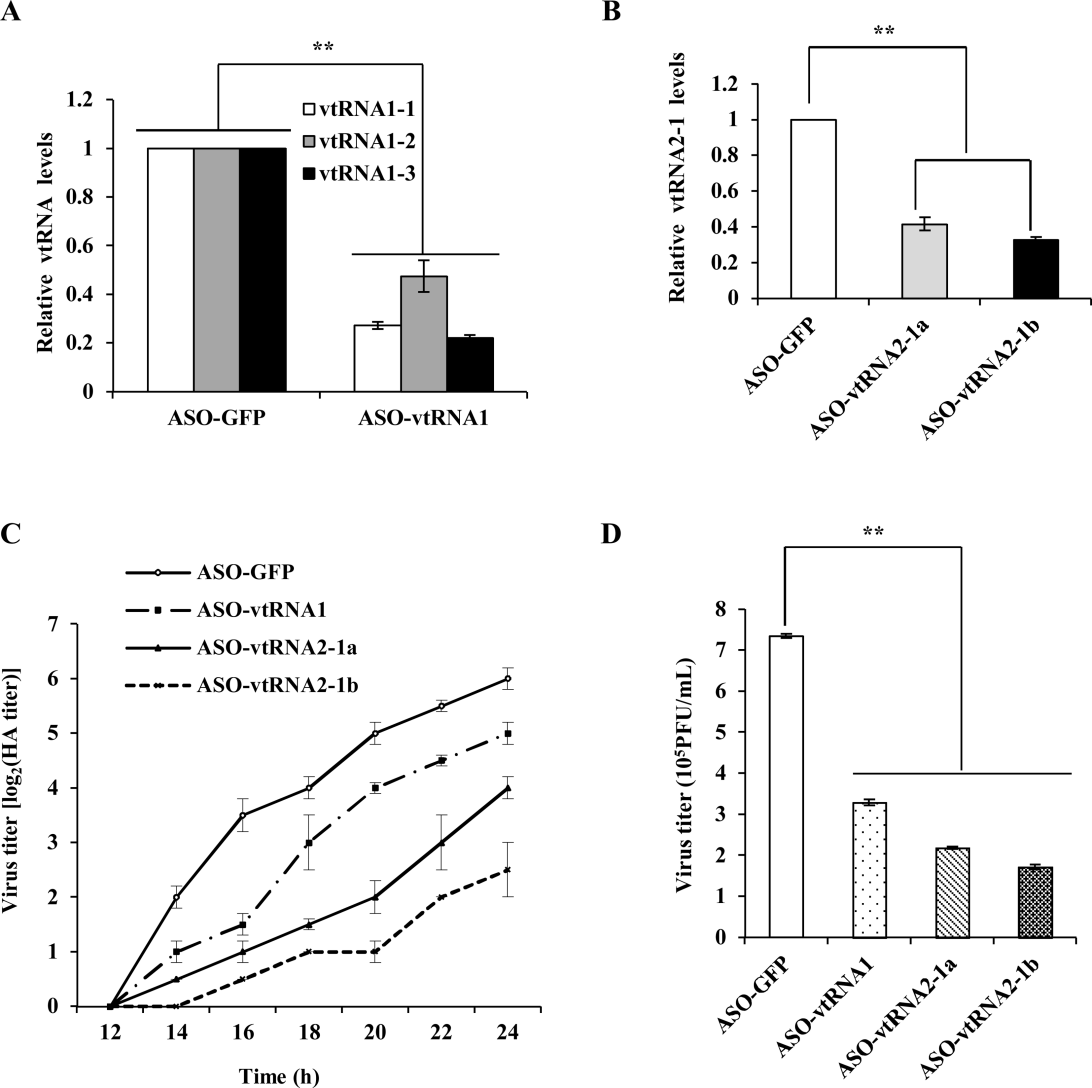
Knockdown of vtRNAs expression significantly impedes IAV replication in A549 cells. (**A**, **B**) A549 cells were transfected with indicated ASOs at 100 nM. 30 h post transfection, cells were infected with WSN for 12 h. The cells were then harvested and the efficiency of ASO-based knockdown of vtRNAs was determined by qRT-PCR. (**C**) A549 cells were transfected with indicated ASOs and then infected with WSN (MOI = 0.5). The supernatants of cell culture were examined for the viral titers by hemagglutinin assay at the indicated time points post infection (p.i.). (**D**) vtRNAs knockdown A549 cells or control cells were infected with WSN as described in (C). Viral titers in the supernatants of these cells were examined by plaque assay (18 h p.i.). Plotted are the average levels from three independent experiments. The error bars represent the SE, **P < 0.01.

Because IAV significantly induced the expression of vtRNAs in host, we hypothesized that vtRNAs might play a critical role in IAV replication. To test this hypothesis, the vtRNAs were silenced by transfecting chemically modified antisense oligonucleotides (ASOs) as reported previously (36). The cells were then infected with or without WSN and harvested at indicated time points post-infection. In consistence with the previous studies (36), the vtRNAs levels were significantly reduced by treatment with ASOs measured via RT-PCR, qRT-PCR or Northern blotting (Figure 2A, B, Supplementary Figure S2A, B, C and D). Remarkably, downregulation of the vtRNAs signiﬁcantly inhibited the viral replication, as detected by hemagglutinin assay (Figure 2C and Supplementary Figure S2E). Silencing vtRNA2-1 had more profound inhibitory effect on IAV replication than silencing vtRNA1 (vtRNA1-1, vtRNA1-2 and vtRNA1-3). These observations were further verified by plaque forming assay (Figure 2D).

**Figure 3.**
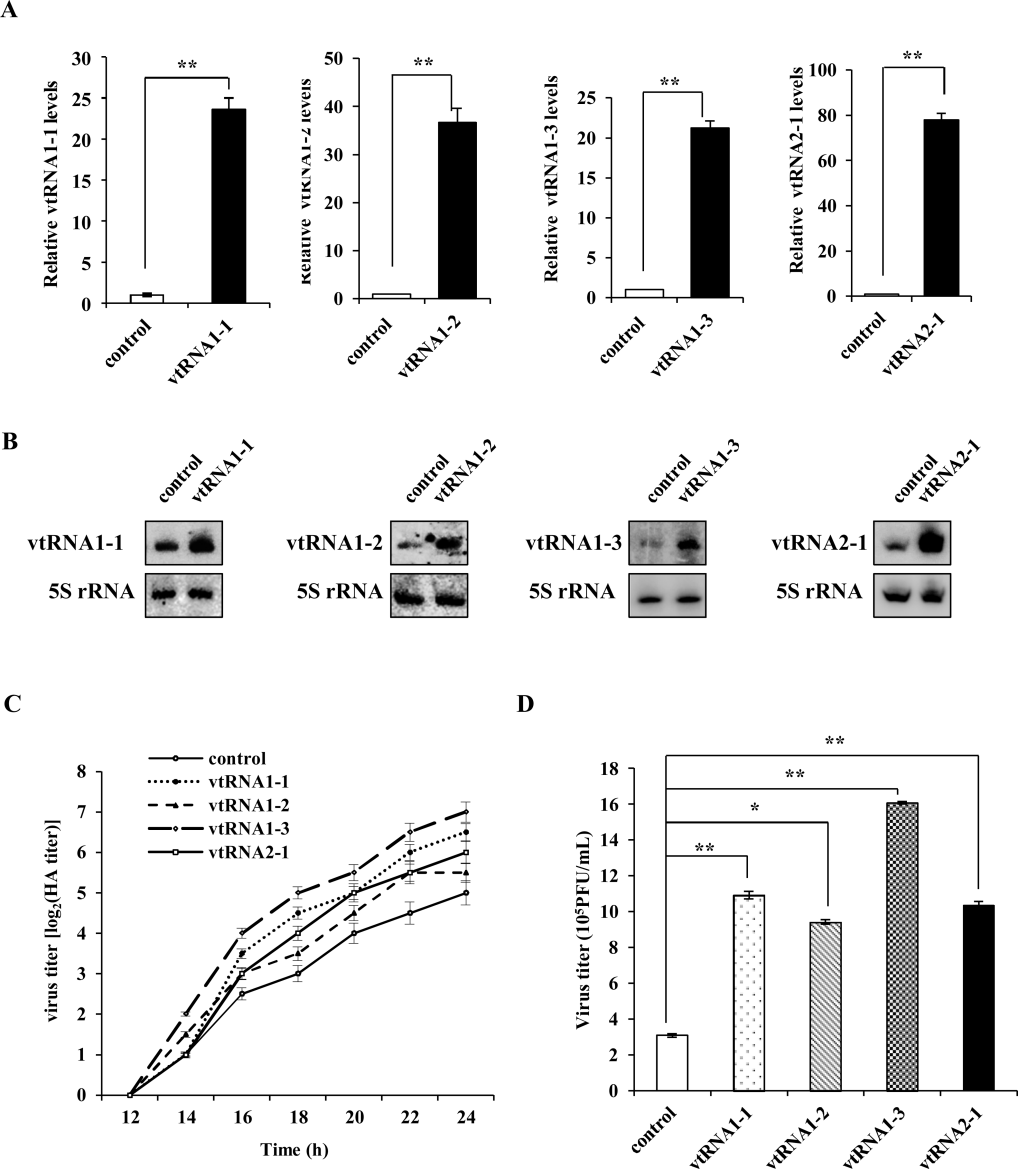
Forced expression of vtRNAs significantly promotes IAV replication in A549 cells. (**A**) Ectopic overexpression of vtRNAs in A549 cells was examined by qRT-PCR. (**B**) Total RNA was extracted from A549 cells overexpressing vtRNAs or empty vector (control). Northern blotting was performed to determine the expression of vtRNAs. (**C**) A549 cells overexpressing vtRNAs or empty vector (control) were infected with WSN (MOI = 0.1). Effects of vtRNA overexpression on IAV replication kinetics in A549 cells were examined by hemagglutinin assay at indicated time points post infection. (**D**) A549 cells overexpressing vtRNAs or empty vector were infected with WSN as described in (C). Viral titers in the supernatants of the cell culture were examined by plaque assay (18 h p.i.). Plotted are the average levels from three independent experiments. The error bars represent the SE, *P < 0.05; **P < 0.01.

To further confirm the functional relevance of vtRNAs in IAV replication, we generated A549 cell lines stably overexpressing four vtRNA members respectively by using a lentiviral expression system (Figure 3A, B, Supplementary Figure S3A and B). Both hemagglutinin assay and plaque assay showed that forced expression of each vtRNA resulted in a signiﬁcant promotion of the viral replication (Figure 3C, D and Supplementary Figure S3C). Taken together, these data suggest that vtRNAs positively regulate the replication of inﬂuenza virus in host, and the induction of vtRNAs in infected cells might be manipulated by the viruses to support their own reproduction.

### Silencing mvtRNA expression in mice significantly enhances resistance of the animals to IAV infection

**Figure 4.**
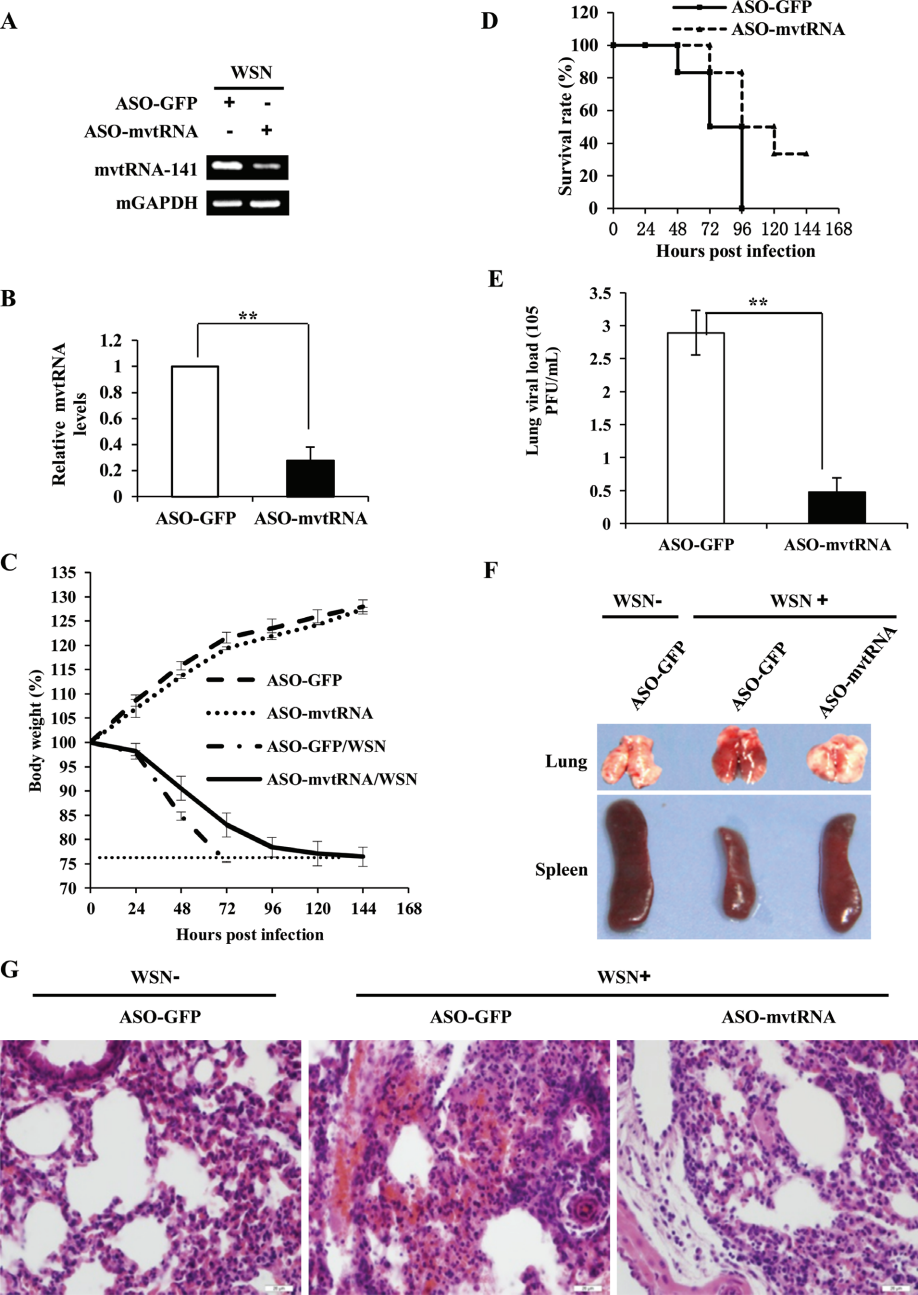
Silencing mvtRNA by inhaled aerosolized ASOs decreases the susceptibility of mice to IAV infection. (**A**, **B**) The C57BL/6 mice that inhaled aerosolized ASOs targeting mvtRNA or GFP for 24 h were infected with WSN (1 × 10^4^ PFU) for another 96 h. Then mice were sacrificed and the lungs were dissected and lysed. The efficiency of ASO-based knockdown of mvtRNA was determined by RT-PCR (A) and qRT-PCR (B). Plotted are the average levels from three independent experiments. The error bars represent the SE, *P < 0.05. (**C**) Shown is the body weight change of mvtRNA knockdown and control mice intranasally inoculated with WSN or PBS (control). Body weight was measured every 24 h. The dashed line indicates the endpoint of 25% weight loss. (**D**) Shown are survival rates of mvtRNA knockdown mice and control mice inoculated intranasally with WSN (8–10 mice/group). Mice were monitored for a period of 144 h. (**E**) Shown are viral titers in the lungs of mvtRNA knockdown and control mice inoculated intranasally with WSN for 96 h. The viral titer was measured by plaque assay. Plotted are the average levels from three independent experiments. The error bars represent the SE, **P < 0.01. (**F**) mvtRNA knockdown and control mice were intranasally inoculated with WSN or PBS for 96 h. Then mice were sacrificed and the lungs and spleens were collected. Shown are representative images from three independent experiments. (**G**) Experiments were performed as described in (F). Shown are representative micrographs of lung sections of the musvtRNA knockdown and control mice stained with hematoxylin and eosin (HE).

It is reported that respirable ASOs represent a novel therapeutic approach for the treatment of lung diseases (46,47). To further define the functional relevance of vtRNA to IAV infection *in vivo*, C57BL/6 mice were treated with aerosolized ASOs targeting mvtRNA (ASO-mvtRNA) or GFP (ASO-GFP) for 24 h, and then infected with WSN for 96 h. As shown in Figure 4A and B, ASO-mvtRNA significantly downregulated mvtRNA expression in lungs of the mice, as compared with the ASO-GFP control. The mvtRNA knockdown mice displayed slower body weight loss and higher survival rate than the control group during viral infection (Figure 4C and D). All control mice died within 96 h p.i., whereas ∼33% of mvtRNA knockdown mice remained alive within 120 h p.i.. Consistent with these observations, the plaque assay indicated that the viral titers in lung tissues of mvtRNA knockdown mice were significantly lower than that in the control group (Figure 4E), suggesting that silencing mvtRNA inhibits the IAV replication *in vivo*. In addition, mvtRNA knockdown mice exhibited less-severe organ damage caused by IAV infection, while the control group showed a greater degree of acute lung injury and spleen atrophy (Figure 4F). Consistently, pathologic examination by hematoxylin and eosin (HE) staining displayed less severe edema and reduced infiltration of inflammatory cells across the interalveolar septum in the lungs of infected mice treated with ASOs targeting mvtRNA (Figure 4G). These results indicate that disruption of mvtRNA expression in mice decreases the susceptibility of the animals to IAV infection.

### NS1 is critically required for IAV-induced expression of vtRNAs *in vitro* and *in vivo*

**Figure 5.**
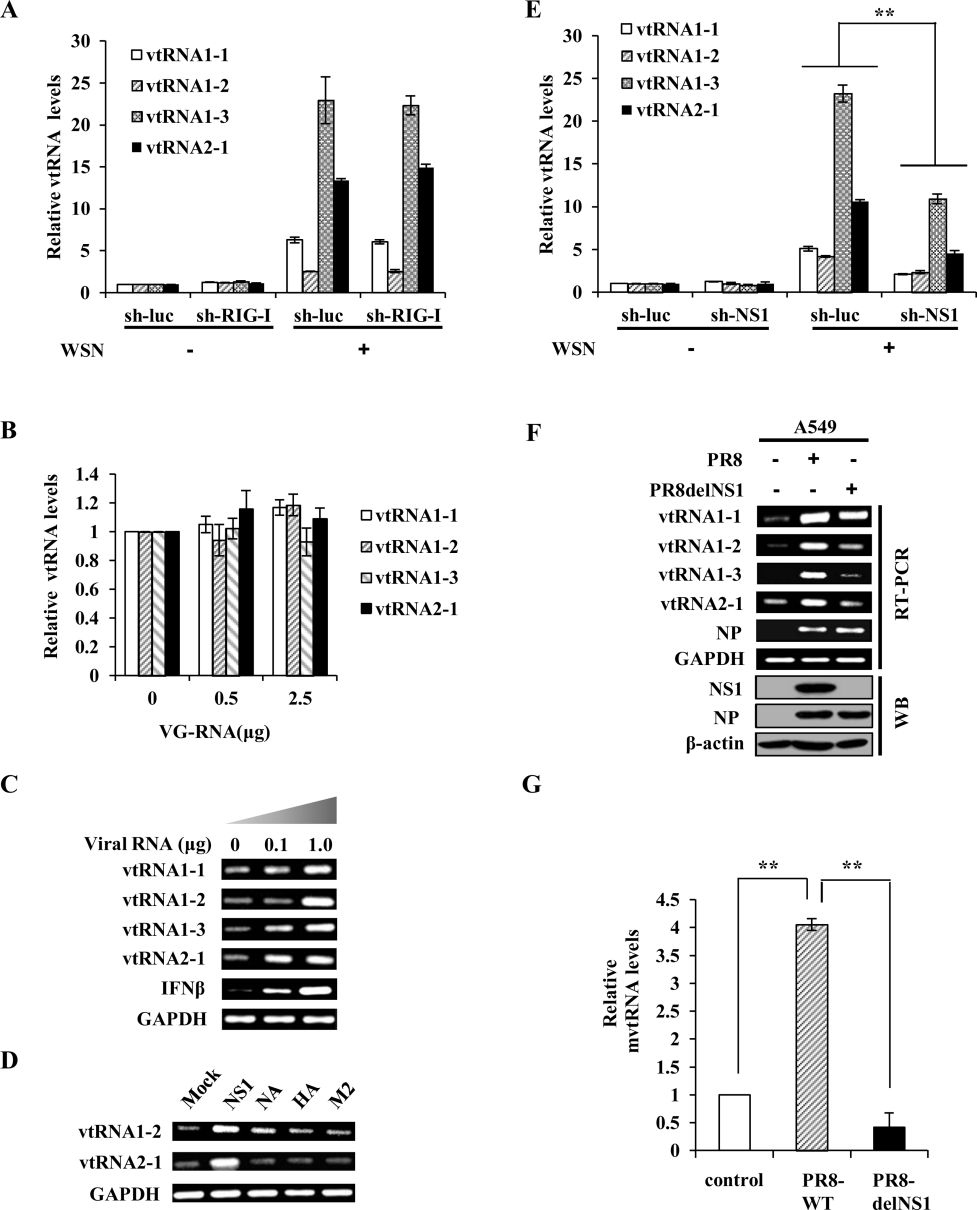
NS1 is required for robust expression of vtRNAs induced during IAV infection both *in vitro* and *in vivo*. (**A**) A549 cells expressing shRNAs targeting RIG-I or luciferase (luc) were infected with or without WSN for 14 h, and then the expression of vtRNAs was examined by qRT-PCR. (**B**) A549 cells were transfected with indicated amount of WSN genomic RNA (VG-RNA) using Lipofectamine 2000. Effect of VG-RNA on the expression of vtRNAs was determined by qRT-PCR. (**C**) Different amounts of total RNA (named as Viral RNA) from A549 cells infected with the IAV were transfected into native A549 cells using Lipofectamine 2000. The expression of vtRNAs in transfected A549 cells was examined by RT-PCR. (**D**) 293T cells were transfected with empty vector (Mock) or plasmids expressing either NS1, NA, M2, or HA protein of IAV using Vigofect. After 36 h post-transfection, the vtRNAs expression was detected by RT-PCR. (**E**) A549 cells stably expressing specific shRNAs targeting NS1 or luciferase (control) were infected with or without WSN. Subsequently, the RNA levels of vtRNAs were measured by qRT-PCR. (**F**) A549 cells were infected with or without PR8 wild-type (WT) or deltaNS1 (delNS1) viruses for 20 h and analyzed by Western blotting and RT-PCR to detect the levels of the indicated genes and proteins. (**G**) Mice intranasally infected with or without PR8 WT or delNS1 viruses for 2 days were sacrificed, and the lungs were dissected and homogenized, followed by qRT-PCR. Shown are representative RT-PCR data from three independent experiments. Plotted are the average levels from three independent experiments. The error bars represent the SE, **P < 0.01.

Next, we determined the inducer of vtRNA expression during the IAV infection. Because vtRNAs act to promote IAV replication, we speculated that the high induction of vtRNAs in IAV-infected cells might not be a host innate defense against the viral infection. Indeed, the levels of vtRNAs were not significantly affected by knockdown of retinoic acid-inducing gene 1 protein (RIG-I), the most important pattern recognition receptor in innate immunity for sensing IAV infection (48) (Figure 5A and Supplementary Figure S4A). To investigate whether viral RNA is the factor responsible for induction of vtRNAs, A549 cells were treated with either different doses of genomic RNA directly isolated from the viruses (Figure 5B, Supplementary Figure S4B and C) or total RNA derived from IAV-infected cells (Figure 5C). We found that the viral RNA isolated from IAV-infected cells but not the viral genome RNA triggered the vtRNA expression. Because the IAV genome consists of eight segments of negative-sense single-stranded RNA (ssRNA) while samples from IAV-infected cells contain viral dsRNA products during IAV replication (41), we next asked if dsRNA could stimulate vtRNA production in A549 cells by treatment with poly (I:C), a dsRNA-mimic. To our surprise, treatment with poly (I:C) failed to induce vtRNA expression (Supplementary Figure S4D). Given that the viral RNA isolated from IAV-infected cells can be translated when transfected into native A549 cells, the induction of vtRNAs may be due to the translated viral proteins. To address this possibility, host cells were transfected with plasmids encoding several different WSN viral proteins. Interestingly, overexpression of NS1, but not hemagglutinin (HA), neuraminidase (NA), and matrix protein 2 (M2), contributed to the significant upregulation of vtRNAs (Figure 5D, and Supplementary Figure S4E). Furthermore, we observed that vtRNAs could be induced not only by NS1 of WSN, but also by NS1 of other IAV strains, including PR8, H7N9, and H5N1 (Supplementary Figure S4F). To confirm this observation, A549 cell lines expressing speciﬁc shRNAs targeting WSN NS1 and luciferase (control) were generated, respectively, as previously described (42). As expected, the elevated levels of vtRNAs were dramatically reduced in NS1 knockdown cells infected with WSN (Figure 5E, and Supplementary Figure S4G). Consistent with these results, the ability of delNS1 A/PR/8/34 virus to induce vtRNAs expression was significantly attenuated both *in vitro* and *in vivo* (Figure 5F, G and Supplementary Figure S4H). Together, our experiments demonstrate that inﬂuenza virus protein NS1 is responsible for the induction of vtRNAs in host.

### vtRNAs inhibit PKR activation and the subsequent interferon expression during the IAV infection

**Figure 6.**
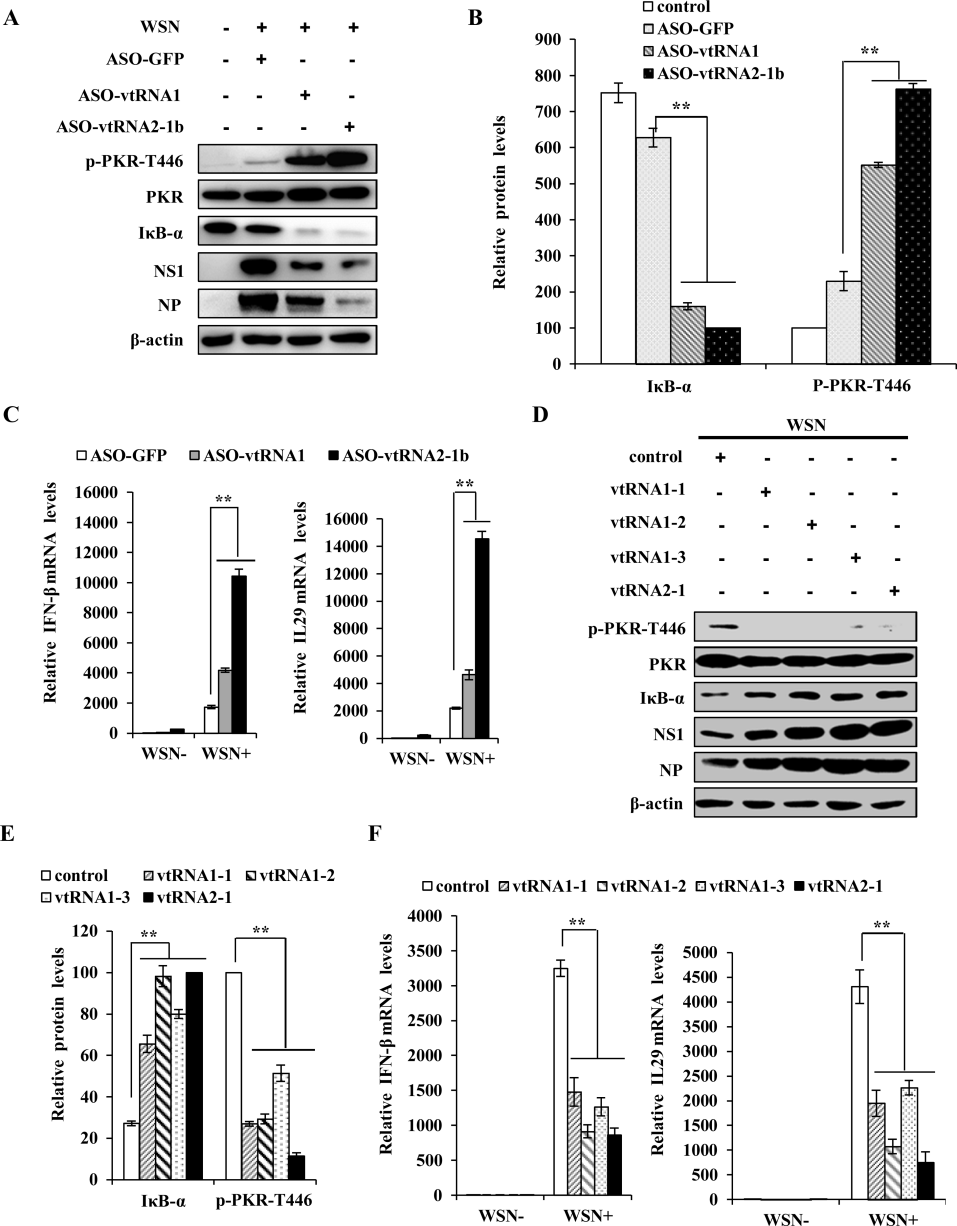
vtRNAs inhibit PKR activation and interferon expression during the IAV infection. (**A**) A549 cells were transfected with indicated ASOs and the samples were then infected with WSN and examined by Western blotting at 14 h p.i. with the indicated antibodies. Shown are representative results from three independent experiments. (**B**) The levels of IκB-α and p-PKR-T466 in (A) were quantitated by densitometry, and normalized to β-actin and PKR expression levels. In each experiment, the lowest levels of IκB-α and p-PKR-T466 are set to 100. Plotted are the average levels from three independent experiments. (**C**) The mRNA levels of IFN-β and IL29 in vtRNAs knockdown cells and control cells infected with or without WSN were determined by qRT-PCR. Plotted are the average levels from three independent experiments. The error bars represent the SE, **P < 0.01. (**D**, **E**) A549 cells stably expressing specific vtRNAs or empty vector were infected with WSN as described in (A). The levels of the indicated proteins were detected by western blotting (D) and quantitated by densitometry (E). (**F**) The mRNA levels of IFN-β and IL29 in vtRNA-overexpressing cells or control cells were determined by qRT-PCR as described in (C).

Previous studies have suggested that vtRNA2–1 can directly bind to PKR and thereby inhibits its activation in HeLa cells (36,49). Since PKR plays a critical role in the host innate defense against viral infection, it is possible that IAV-induced vtRNA2-1 promotes the viral replication by inhibiting PKR activation. To explore whether induced vtRNAs affect activation of PKR signaling, vtRNA1 and vtRNA2-1 were downregulated by using ASO-vtRNA1 and ASO-vtRNA2-1b, respectively. Indeed, silencing vtRNA1 or vtRNA2–1 expression resulted in a marked increase in the level of PKR phosphorylation (Thr446), but dramatically reduced the level of NF-κB inhibitor IκB-α during the IAV infection (Figure 6A and B). In contrast, co-transfection of cells with control ASO-GFP and GFP-expressing vector had no significant effect on PKR activity (Supplementary Figure S5A), indicating that it is the reduction of vtRNAs rather than the formation of ASO:vtRNA dsRNA that is responsible for the activation of PKR in IAV infected host. In non-infected cells, knockdown of vtRNA1 or vtRNA2–1 by ASOs also caused an increase in phosphorylated PKR (Supplementary Figure S5B), suggesting that vtRNAs normally function as inhibitors of PKR activation in cells. Since reduced IκB-α level indicates the activation of NF-κB signaling and subsequent interferon response (3), we examined whether altering the expression of vtRNAs had any effects on the production of type I interferon IFN-β and type III interferon IL29. Using qRT-PCR assay, we found that expression of these interferons was significantly upregulated upon depletion of vtRNAs (Figure 6C). To verify these findings, A549 cell lines stably overexpressing vtRNAs were used. As expected, ectopic expression of vtRNAs attenuated the PKR and NF-κB signaling, and concurrently inhibited the expression of IFN-β and IL29 (Figure 6D–F). In addition, we found that level of NF-κB inhibitor IκB-α was reduced in lungs of mvtRNA knockdown mice infected with WSN (Supplementary Figure S5C). Together, these data suggest that IAV-triggered expression of vtRNAs enhances the viral replication through suppression of PKR and the subsequent interferon production.

### IAV NS1 suppresses PKR activation through increased vtRNAs in the infected cells

**Figure 7.**
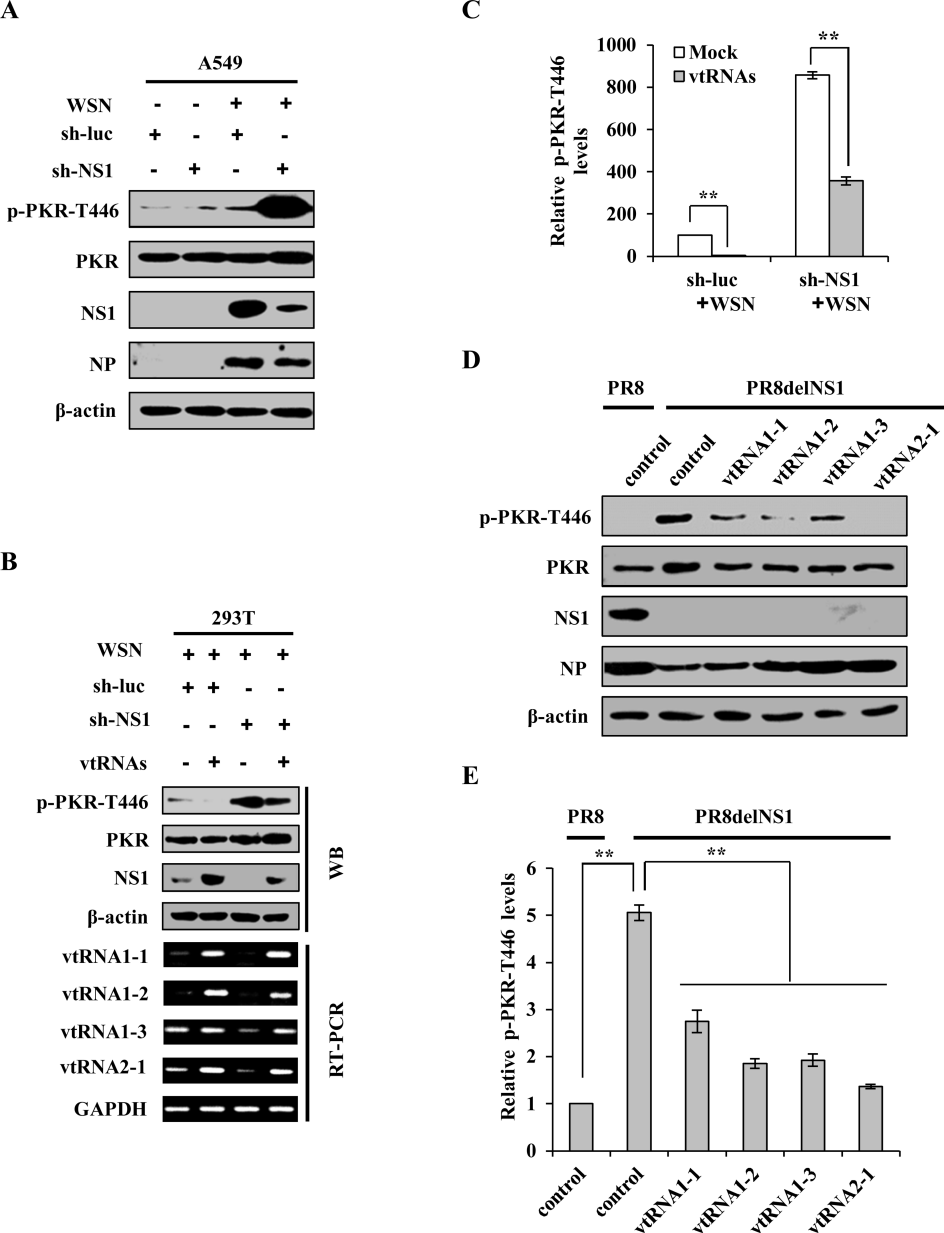
NS1 suppresses PKR activation through increased expression of vtRNAs in IAV-infected cells. (**A**) A549 cell lines stably expressing specific shRNAs targeting NS1 or luciferase (control) were infected with or without WSN and harvested at 12 h p.i., followed by analysis of Western blotting using the indicated antibodies. Shown are representative data from three independent experiments with similar results. (**B**) Rescue assay was performed. 293T cells co-transfected with plasmids encoding empty vector (Mock), or vtRNAs, and speciﬁc shRNAs targeting NS1 or luciferase (control) were infected with WSN for 12 h, followed by RT-PCR and western blotting using the indicated antibodies. Shown are representative blots from three independent experiments with similar results. (**C**) The p-PKR-T466 levels shown in (B) were quantitated by densitometry and normalized to β-actin levels as described in Figure 6B. In each experiment, the p-PKR-T466 level in infected cells co-transfected with plasmids encoding empty vector and speciﬁc shRNAs targeting luciferase (control) is set to 100. Plotted are the average levels from three independent experiments. The error bars represent the SE, **P < 0.01. (**D**) A549 cell lines stably overexpressing vtRNAs and control cells were infected with PR8 WT or delNS1 viruses and harvested at 14 h p.i., followed by Western blotting using the indicated antibodies. Shown are representative blots from three experiments with similar results. (**E**) The p-PKR-T466 levels shown in (D) were quantitated by densitometry and normalized to β-actin levels. In each experiment, the p-PKR-T466 level in control cells infected by PR8 WT is set to 100. Plotted are the average levels from three independent experiments. The error bars represent the SE, **P < 0.01.

Our data presented above show that vtRNAs are upregulated in a viral NS1-dependent manner in IAV-infected cells and act as inhibitors of PKR. This is consistent with a previous study indicating that PKR is hardly activated upon IAV infection (9). Since it is known that NS1 plays a key role in blocking activation of PKR during IAV infection, likely, IAV NS1 protein might exploit host vtRNAs to inhibit PKR activation. For this, A549 cell lines stably expressing specific shRNAs targeting either NS1 or luciferase (control) were infected with or without WSN and harvested at 12 h p.i., followed by western blotting. We found that silencing NS1 greatly elevated the level of PKR phosphorylation in cells infected with IAV (Figure 7A). Next, we asked whether this was due to the decreased expression of vtRNAs in NS1 knockdown cells. To this end, vtRNAs were overexpressed in NS1 knockdown cells infected with IAV. Of interest, overexpression of vtRNAs significantly reduced the increased PKR phosphorylation caused by NS1 silencing (Figure 7B and C). To further confirm this finding, A/PR/8/34 and delNS1 A/PR/8/34 viruses were used to infect A549 cells stably overexpressing specific vtRNAs. Western blotting analysis demonstrated that forced expression of vtRNA family members significantly impaired the PKR activation in cells infected with delNS1 A/PR/8/34 viruses (Figure 7D and E). These results reveal that increased expression of vtRNAs are required for NS1-mediated suppression of PKR activation in the infected host cells.

### Involvement of vtRNAs in inactivation of PKR signaling by infections of two other viruses

**Figure 8.**
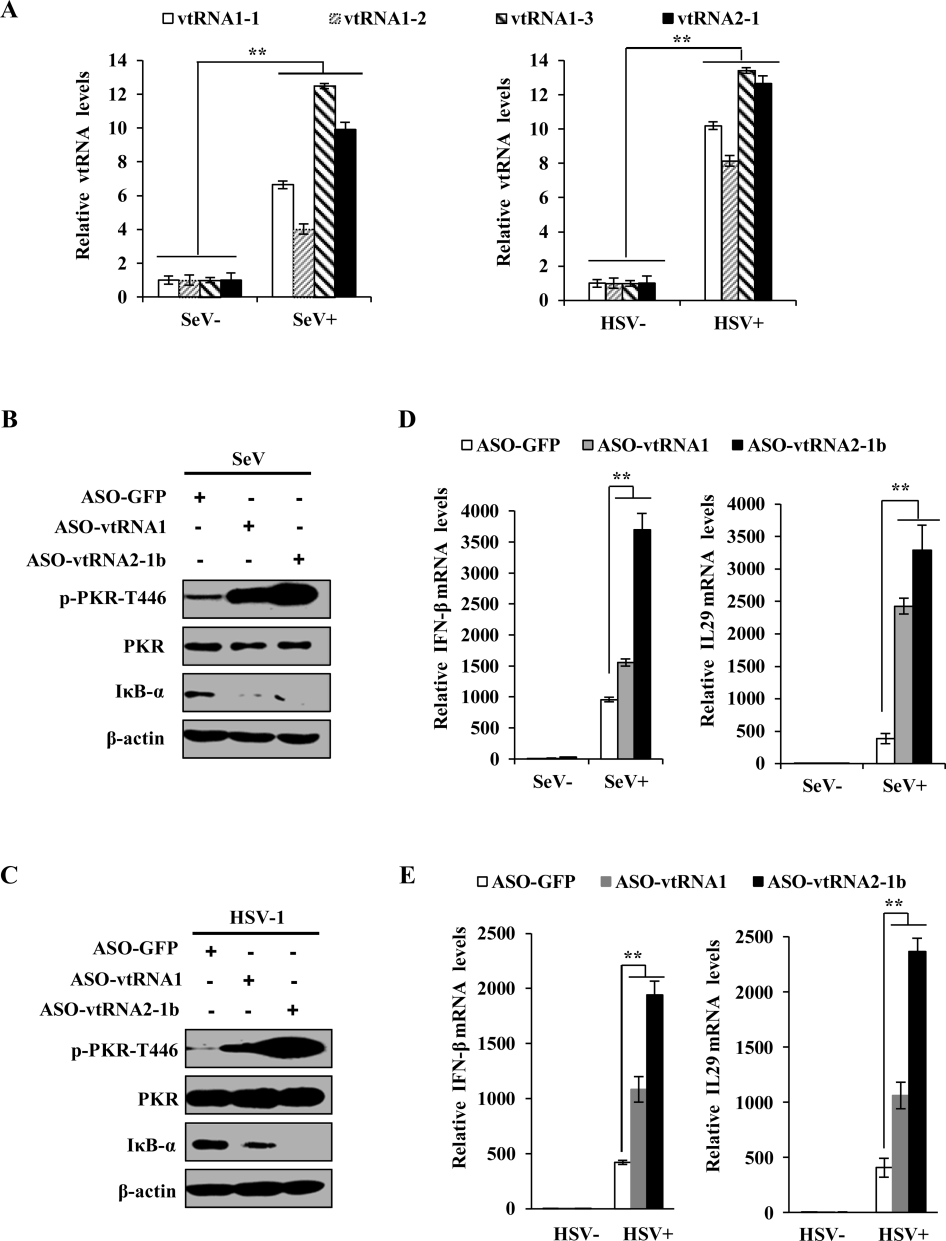
Involvement of vtRNAs in inactivation of PKR signaling by infections of two other viruses. (**A**) A549 cells were infected with Sendai virus (SeV) for 12 h, or herpes simplex virus 1 (HSV-1) for 24 h, and subsequently, the levels of vtRNAs were examined by qRT-PCR. (**B**–**E**) A549 cells transfected with indicated ASOs were infected with SeV or HSV-1 as described in (A), followed by Western blotting (B, C) and qRT-PCR (D, E) to determine the expression of indicated proteins and genes. Shown in (B) and (C) are representative blots from three independent experiments. Plotted are the average levels from three independent experiments in (D) and (E). The error bars represent the SE, **P < 0.01.

Because vtRNAs have been found to be upregulated upon infections with EBV and Kaposi's sarcoma virus (HHV8) (32,35), we asked if more other viruses could also induce expression of vtRNAs. To address this issue, we monitored expression of vtRNAs in cells infected with or without Sendai virus (SeV) and herpes simplex virus 1 (HSV-1). Interestingly, the results from RT-PCR and qRT-PCR showed that infections with these viruses were able to significantly induce vtRNAs expression (Figure 8A and Supplementary Figure S6A). Next, we determined whether the induced vtRNAs were involved in regulating the activation of PKR in cells infected by these viruses. Strikingly, experiments using RNA interference demonstrated that knockdown of vtRNAs greatly increased the phosphorylation levels of PKR, but reduced the levels of IκB in the virus-infected cells (Figure 8B and C). As predicted, the expression of IFN-β and IL29 was dramatically enhanced by silencing the vtRNAs in these infected cells (Figure 8D and E). Taken together, these experiments provide strong evidence that vtRNAs inhibit activation of PKR and IFN production in host during a broad range of viral infections. Therefore, our findings suggest that a variety of viruses may have evolved a strategy to circumvent the host PKR-mediated innate immune response through increasing host vtRNAs.

## DISCUSSION

To complete their life cycle, viruses have developed multiple strategies to antagonize the host antiviral response during the infection, including hijacking or inactivating many host factors and signaling pathways (50–56). As an important sentinel kinase, PKR plays a key role in the innate immune response to viral infection and is strictly controlled by host (57–59). On the other hand, many viruses, including influenza virus, utilize multiple modulations to repress the antiviral effects of PKR (60–62). However, the precise mechanisms by which influenza virus counteracts the PKR-dependent innate immune signaling are not fully understood. The findings in this study have for the first time demonstrated that vtRNAs, as a negative regulator of PKR, are hijacked by influenza virus during infection, which greatly enhances the expression of vtRNAs, thereby inhibiting PKR activity and promoting viral replication in host.

vtRNAs have been recognized as components of the vault complex, which is a gigantic (13 MDa), hollow, oval-shaped cytoplasmic ribonucleoprotein involved in multidrug resistance (31). However, new evidence has revealed that most vtRNAs (95%) are actually not present in the pelleted fraction with vault complex, but exist in free states (32). In human cells, there are four functional vtRNAs: vtRNA1-1, vtRNA1-2, vtRNA1-3 and vtRNA2-1. Our studies revealed that these vtRNAs were expressed in various cell types and their expression was significantly increased upon infection not only by influenza virus, but also by SeV and HSV-1. This is consistent with the previous investigation that vtRNAs are specifically upregulated in γ-herpesviruses infected human lymphocytes (32). Although our experiments and a previous report showed that vtRNAs were under detectable level in 293T cell by analysis of Northern blot (49), vtRNAs could be indeed detected by RT-PCR in this cell type in several independent experiments (Figure 1E and Supplementary Figure S1B). Taken together, these observations provide evidence that there may be a broad range of viruses that can induce robust expression of vtRNAs in a variety of hosts during the viral infection.

In this study, using *in vitro* and *in vivo* analysis, we found that viral protein NS1 of IAV was the key factor to induce the upregulation of vtRNAs. For other viruses like SeV and HSV-1, there might be specific viral proteins that are responsible for the upregulation of vtRNAs during viral infections. This remains to be further determined. As a critical viral protein, NS1 is well-known to have multiple functions to inhibit host immune responses (63), such as evading the recognition of cytosolic viral RNA sensor RIG-I, antagonizing type I IFN-dependent response (53,64), and suppressing the antiviral proteins, including PKR and OAS/RNase L (15,17,18). As to these functions of NS1, some mechanisms are well established, whereas others, such as how NS1 represses the PKR activity, are still not clear. In particular, it is previously unknown whether vtRNAs are regulated by NS1 (18,57). Because the NS1 protein contains an RNA-binding domain, it is possible that NS1 could directly bind and stabilize the vault RNAs. Besides, many studies have shown that host genes have aberrant epigenetic modiﬁcation upon viral infection. For example, the expression of IL-4 receptor and metallothionein-1F are downregulated by promoter methylation induced by Hepatitis B virus X protein via interaction with DNMT3A (65). In addition, it was observed that IL-32 and IL-6 are up-regulated by aberrant epigenetic modiﬁcation during IV infection (66,67). Both our work and previous studies have shown that the expression of vtRNA2–1 was regulated by DNA methylation (Supplementary Figure S7A) (38–39). However, the epigenetic mechanisms underlying regulation of vtRNA expression by NS1 of IAV remains to be further investigated. On the other hand, a recent study has revealed that LMP-1 protein of Epstein–Barr virus can induce expression of vtRNA1–1 through activating transcription factor NF-κB (68). Therefore, in the future studies, it is of interest to determine the transcription factor(s) that regulates the expression of vtRNAs triggered by IAV NS1.

Importantly, we found that elevated vtRNAs promoted the replication of IAV. This indicates that the virus could exploit vtRNAs through NS1 to support viral reproduction. Since vtRNAs are the functional components of the vault complex, we thought that the antiviral effects might be due to the vault complex. However, results from our microarray analysis revealed that the mRNA levels of major protein component of vault complex (major vault protein, MVP) and minor vault protein (telomerase-associated protein 1, TEP1) were downregulated during influenza virus infection (http://www.ncbi.nlm.nih.gov/geo/; GEO access number GSE58741). Experiments using Western blotting further confirmed that the MVP protein level was reduced during the virus infection (Supplementary Figure S8A). Previous observations showed that only 5% of vtRNAs in cells were directly associated with the vault particle (32). Thus, further studies are required to determine whether altered expression of vtRNAs changes the vault complex, which somehow may be important for the effects on PKR activation and influenza virus replication. Recently, Yong and his colleagues have identified that vtRNA2-1 (also named as pre-miR-886 or nc886) is a novel putative tumor suppressor by modulating PKR activity (36–39) and can repress PKR by direct physical interaction (49). Because PKR is a key signaling molecule during antiviral immune response, we postulated that vtRNAs promote virus replication by inhibiting PKR. Through knockdown and overexpression of vtRNAs, we indeed demonstrated that NS1-induced upregulation of vtRNAs was responsible for the inhibition of PKR and the subsequent interferon antiviral response during IAV infection. Similar results were obtained during the infections of other RNA virus (SeV) and DNA virus HSV-1. Although a previous study indicated that vtRNA1–1 in HCT116 cells was not capable to inhibit PKR activation as compared with vtRNA2–1 (36), we found that the simultaneous knockdown of vtRNA 1-1, 1-2 and 1-3 in A549 cells by ASO-vtRNA1 could cause the activation of PKR significantly. This might be due to the additive effects of these vtRNAs, or the different intracellular environments between HCT116 (human colorectal carcinoma cell) and A549 (human lung cancer cell) that might affect the vtRNA functioning. Furthermore, our *in vivo* studies also showed that mvtRNA knockdown mice exhibited decreased susceptibility to influenza virus infection, as evidenced by reduced viral replication in lung, attenuated acute lung injury and spleen atrophy, and consequently increased survival rates. However, the physical interaction between mouse vtRNA and PKR needs to be clarified in the future. Together, these data suggest that utilization of host vtRNAs by viruses to inhibit PKR signaling pathway might be an important and universal strategy to antagonize the host innate immunity.

In conclusion, here we have identified a novel mechanism by which IAV escapes the PKR-mediated antiviral response by upregulating vtRNAs through viral protein NS1. We postulate that under normal physiological conditions, PKR activity is well regulated by vtRNAs to maintain the cell homeostasis; but under virus infection conditions, vtRNAs can be exploited by viruses to impair the PKR-dependent antiviral response, thereby promoting efficient viral replication in host cells. Our findings unveil a novel strategy for viruses to antagonize host innate immunity. The results also present evidence to explain how NS1 inhibits the antiviral function of PKR during IAV infection. Further investigations are needed to explore the relationship between vtRNAs and more other viruses and identify the exact proteins in these viruses that regulate the expression of vtRNAs. These studies will provide novel insights into the complex mechanisms of how viruses counteract host antiviral immunity.

## ACCESSION NUMBER

The Gene Expression Omnibus (GEO) accession number for the microarray data reported in this paper is GSE58741 (www.ncbi.nlm.nih.gov/geo/).

## Supplementary Material

SUPPLEMENTARY DATA
