# Supplementary material for: Robust expression of vault RNAs induced by influenza A virus plays a critical role in suppression of PKR-mediated innate immunity
Source: Nucleic Acids Res. 2015 Oct 20;43(21):10321–37. doi: 10.1093/nar/gkv1078 (PMC4666359; doi:10.1093/nar/gkv1078)
Supplement: SUPPLEMENTARY DATA [file supp_43_21_10321__index.html]

Robust expression of vault RNAs induced by influenza A virus plays a critical role in suppression of PKR-mediated innate immunity — SUPPLEMENTARY DATA 

# Robust expression of vault RNAs induced by influenza A virus plays a critical role in suppression of PKR-mediated innate immunity

## SUPPLEMENTARY DATA

- SUPPLEMENTARY DATA
