# Supplementary material for: Robust expression of vault RNAs induced by influenza A virus plays a critical role in suppression of PKR-mediated innate immunity
Source: Nucleic Acids Res. 2015 Oct 20;43(21):10321–37. doi: 10.1093/nar/gkv1078 (PMC4666359; doi:10.1093/nar/gkv1078)

Supplementary Materials

Supplementary Figure Legends

Figure S1. Expression of vtRNAs is significantly upregulated during IAV infection, related to figure 1. (A) A549 cells were infected with or without WSN for 16 h. RT-PCR was performed to determine the expression of vtRNAs. (B) The expression of vtRNA2-1 in WSN-infected or uninfected 293T and A549 cells (16 hpi) were determined by RT-PCR with or without reverse transcriptase. (C) A549 cells were infected with WSN at indicated MOIs for 16 h. qRT-PCR was performed to determine vtRNAs expression. (D) The expression of vtRNAs in indicated human cell lines infected with or without WSN for 16 h was examined by Northern blotting. 5S rRNA was used as a loading control. (E) The mvtRNA expression in mouse cell lines infected with or without WSN for 18 h was examined by RT-PCR. The viral nucleoprotein (NP) was examined by Western blotting. (F, G) C57BL/6 mice intranasally infected with or without WSN (5×10^4 PFU) for 2 days were sacrificed, and the mvtRNA in the lungs were then examined by RT-PCR with or without reverse transcriptase (F) or qRT-PCR (G). (H, I) C57BL/6 mice infected with or without WSN (5×10^4 PFU) for indicated time. The mvtRNA in the lung were examined by RT-PCR using indicated primers. (J) Shown are the cDNA sequences of mvtRNA-251 amplicon in (I), which was RT-PCR product (251 nt long) from primer pair located outside of the 141 nt region based on Vaultrc5 in the database. The bold underlined sequences are the 141 nt transcript. Shown are representative results from three independent experiments. The error bars represent the S.E., $^{**}P < 0.01$, $^{*}P < 0.05$.

Figure S2. Altering expression of vtRNAs affects IAV replication in A549 cells, related to figure 2. (A-C) A549 cells were transfected with indicated ASOs at 100 nM for 30 hours and infected with WSN for 12 h. The knockdown efficiency of vtRNAs was determined by RT-PCR (A, B) and Northern blotting (C). (D) The vtRNA levels shown in (C) were quantitated by densitometry and normalized to 5S rRNA levels. In each experiment, the vtRNA levels in control (ASO-GFP) is set to 100. (E) A549 cells transfected with ASOs targeting vtRNAs were infected with WSN. The virus titers in the supernatants of cell culture were examined by hemagglutinin assay at the indicated time points post infection (p.i.). Plotted are the average levels from three independent experiments. The error bars represent the S.E., $^{**}P < 0.01$, $^{*}P < 0.05$.

Figure S3. Forced expression of vtRNAs significantly promotes IAV replication in A549 cells, related to figure 3. (A) Lentiviruses encoding indicated vtRNAs or control were produced in 293T cells. Cell culture supernatants containing lentiviruses were collected and filtered through a 0.22-μm MCE membrane (Millipore). A549 cells were then infected with these lentiviruses to generate specific cell lines as indicated. Shown are micrographs obtained from a fluorescent microscope (Axiovert 200M; Zeiss, Oberkochen, Germany). (B) Ectopic overexpression of vtRNAs in A549 cells was examined by RT-PCR. (C) A549 cells overexpressing vtRNAs or empty vector control were infected with WSN. The virus titers in the supernatants of cell culture were examined by hemagglutinin assay at the indicated time points post infection (p.i.).

Figure S4. NS1 but not RIG-I-dependent signaling is required for IAV-induced expression of vtRNAs *in vitro* and *in vivo*, related to figure 5. (A) A549 cells expressing shRNAs targeting RIG-I or luciferase (luc) were infected with or without WSN for 14 h, and then the expression of vtRNAs was examined by RT-PCR. (B) A549 cells were transfected with indicated amount of WSN genomic RNA (VG-RNA) as previously described in Figure. 5B, followed by RT-PCR to detect vtRNA levels. (C) Experiments were performed as described in (B). IFN-β mRNA levels were examined by qRT-PCR. (D) Different amounts of poly (I:C) were transfected into A549 cells using Lipofectamine 2000. The expression of vtRNAs in transfected A549 cells was examined by RT-PCR. (E) 293T cells were transfected with indicated amount of plasmid expressing IAV NS1. The expression of vtRNAs in transfected cells was examined by RT-PCR. (F) A549 cells were transfected with plasmids expressing NS1 proteins of different IAV strains including WSN (H1N1), PR8 (H1N1), H7N9 (A/chicken/Wuxi/2013) and H5N1 (A/bar-headed goose/Qinghai/2005). The expression of vtRNAs in transfected cells was examined by RT-PCR. The NS1 mRNA levels were detected by RT-PCR using respective primers for NS1 from different IAV strains. (G) A549 cells stably expressing specific shRNAs targeting NS1 or luciferase (control) were infected with or without WSN. Subsequently, the cell lysates were analyzed by Western blot probed with indicated antibodies, and the RNA levels of vtRNAs were measured by RT-PCR. (H) Mice were intranasally infected with or without PR8 WT or delNS1 viruses for 2 days. RT-PCR was performed to detect the mvtRNA levels in lungs. Shown are representative RT-PCR data from three independent experiments.

Figure S5. Silencing vtRNAs expression activates PKR phosphorylation and signaling, related to figure 6. (A) 293T cells were co-transfected with ASO-GFP and/or GFP-expressing vector for 42 h. The PKR phosphorylation (Thr446) level in these cells was examined by Western blotting. (B) A549 cells were transfected with indicated ASOs for 14 h. The PKR phosphorylation (Thr446) level, total PKR protein level and IκB-α level were examined by Western blotting. (C) The C57BL/6 mice that inhaled aerosolized ASOs targeting mvtRNA or GFP for 24 h were then infected with WSN (1×10^4 PFU) for 72 h. The mice were sacrificed and the lungs were dissected and lysed, followed by Western blotting with indicated antibodies to determine viral protein NP and IκB-α. Shown are representative results from three independent experiments.

Figure S6. vtRNAs is upregulated during SeV and HSV-1 infection, related to figure 8. (A) A549 cells were infected with sendai virus (SeV) for 12 h or herpes simplex virus 1 (HSV-1) for 24 h. The levels of vtRNAs were examined by RT-PCR. Shown are representative results from three independent experiments.

Figure S7. Expression of vtRNA2-1 is likely regulated by DNA methylation. (A) A549 cells were treated with or without 10 μM DNA methyltransferase inhibitor 5-Aza-2'-deoxycytidine (decitabine; DAC, Sigma, USA) for 2 days, and supplemented with fresh DAC every 24h. Then cells were infected with or without WSN for 18 h. The expression of vtRNA2-1 was examined by RT-PCR. IL6 was used as a positive control. Shown are representative results from three independent experiments.

Figure S8. MVP protein level was reduced during IAV infection. (A) A549 cells were infected with or without WSN for 18 h, and the levels of MVP were examined by Western blotting. Shown are representative results from three independent experiments.

Figure S1

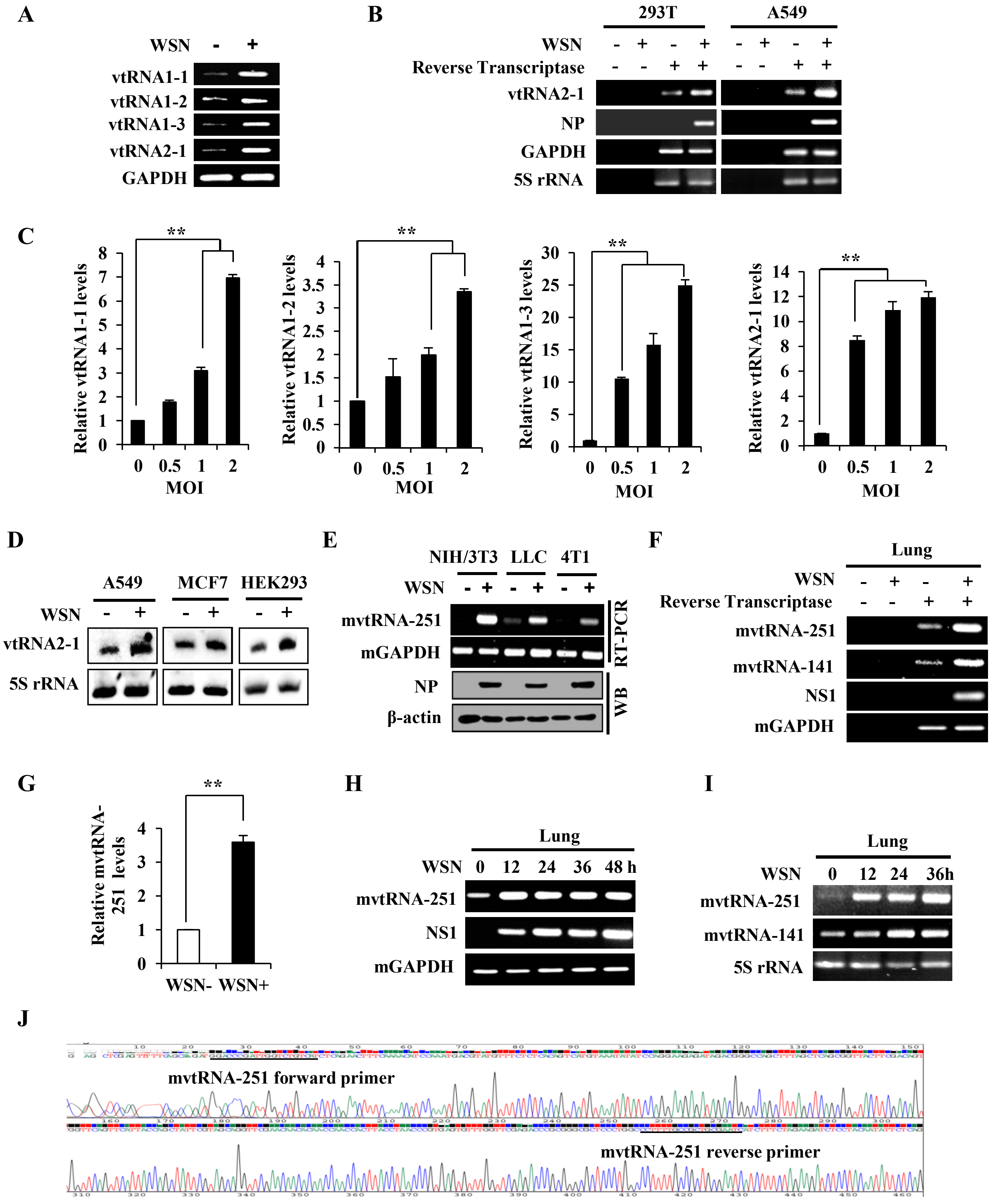

GGACCCGATTGGTCTGTCATCTCAGAACTTTCAAAACATCCAAATGACGTATGTTTCTCTCACAGTCATGT
AAATATATCCAGGAAGAGATAGACG**GGCCAGCTTTAGCTCAGCGGTTACTTCGACAGTGGTTCAGTT**
CATTACCAGCTATTCGTAGCAGGTTCGAACAACACAACCAACCACTTACCTAACCCGTGAGTGTTTG
GTTCGAGACCCGCGGGCGCTCCCTGGCCCTTTTGCCGCTGCGAATC

Figure S2

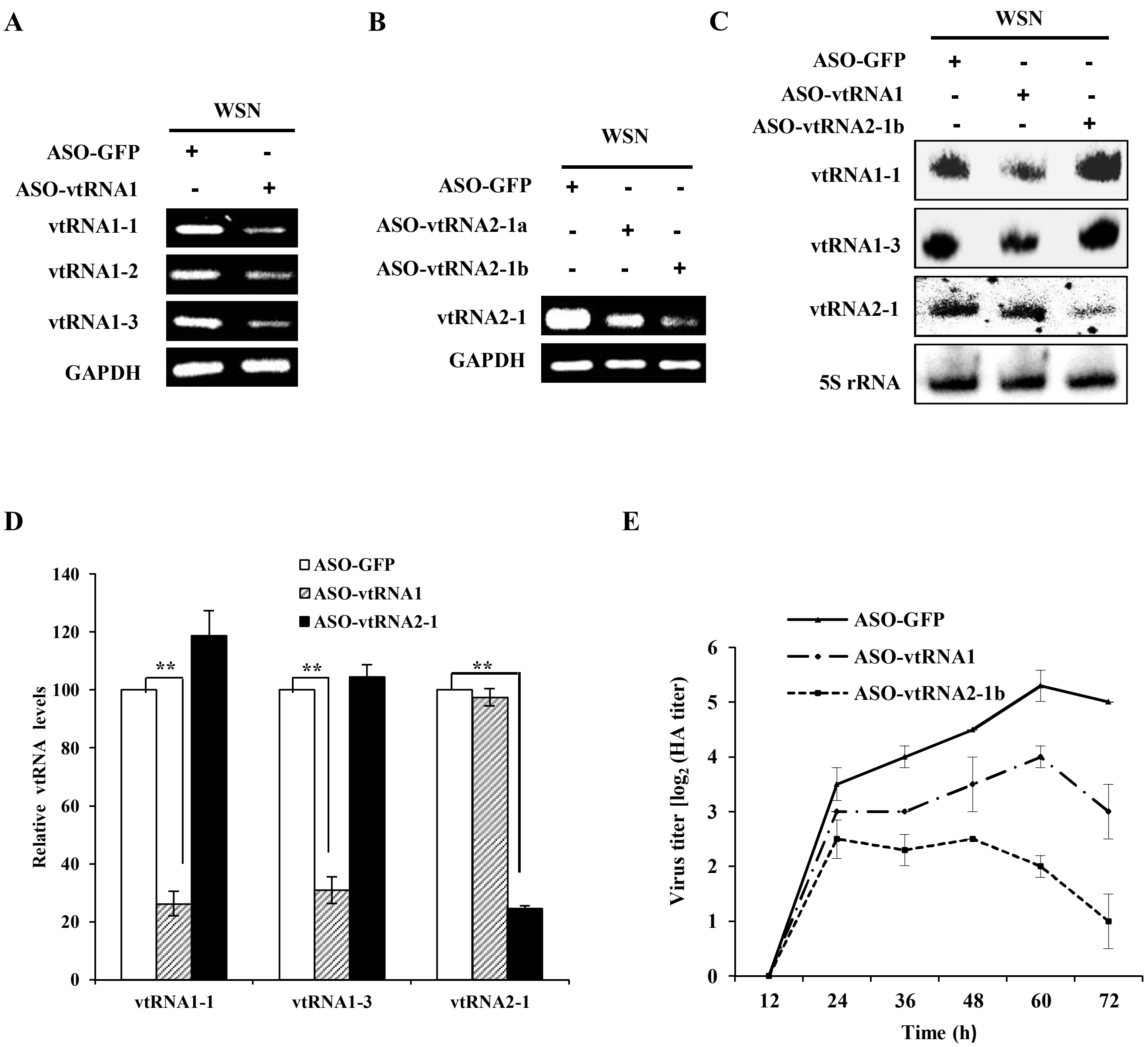
A
WSN
ASO-GFP + -
ASO-vtRNA1 - +
vtRNA1-1
vtRNA1-2
vtRNA1-3
GAPDH
B
WSN
ASO-GFP + - -
ASO-vtRNA2-1a - + -
ASO-vtRNA2-1b - - +
vtRNA2-1
GAPDH
C
WSN
ASO-GFP + - -
ASO-vtRNA1 - + -
ASO-vtRNA2-1b - - +
vtRNA1-1
vtRNA1-3
vtRNA2-1
5S rRNA
D
ASO-GFP
ASO-vtRNA1
ASO-vtRNA2-1
Relative vtRNA levels
0
20
40
60
80
100
120
140
**
**
**
vtRNA1-1
vtRNA1-3
vtRNA2-1
E
ASO-GFP
ASO-vtRNA1
ASO-vtRNA2-1b
Virus titer [log2 (HA titer)
0
1
2
3
4
5
6
12
24
36
48
60
72
Time (h)

Figure S3

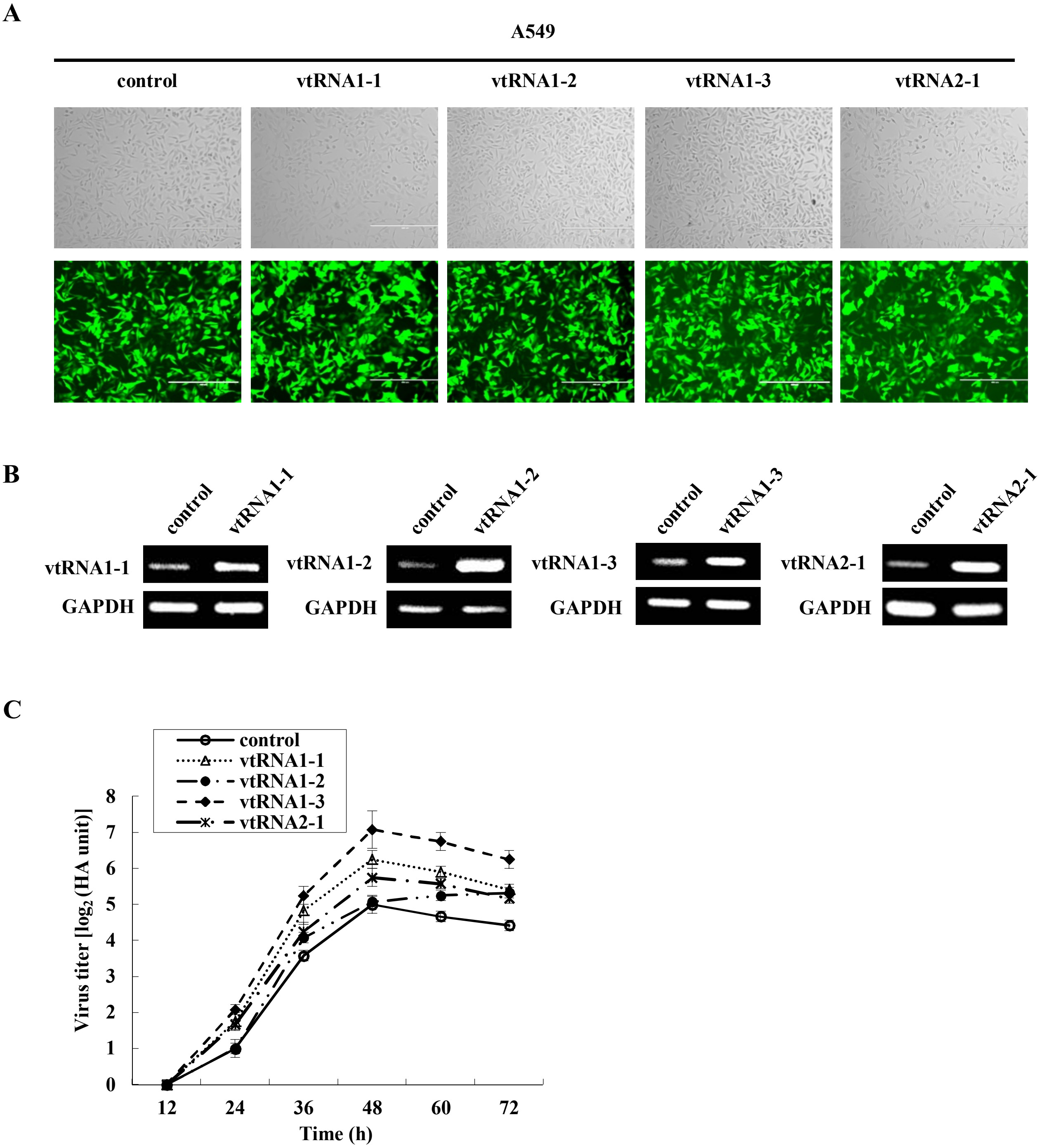
A
A549
control
vtRNA1-1
vtRNA1-2
vtRNA1-3
vtRNA2-1
B
control
vtRNA1-1
vtRNA1-2
vtRNA1-3
vtRNA2-1
GAPDH
C
control
vtRNA1-1
vtRNA1-2
vtRNA1-3
vtRNA2-1
Virus titer [log2 (HA unit)]
0
1
2
3
4
5
6
7
8
12
24
36
48
60
72
Time (h)

Figure S4

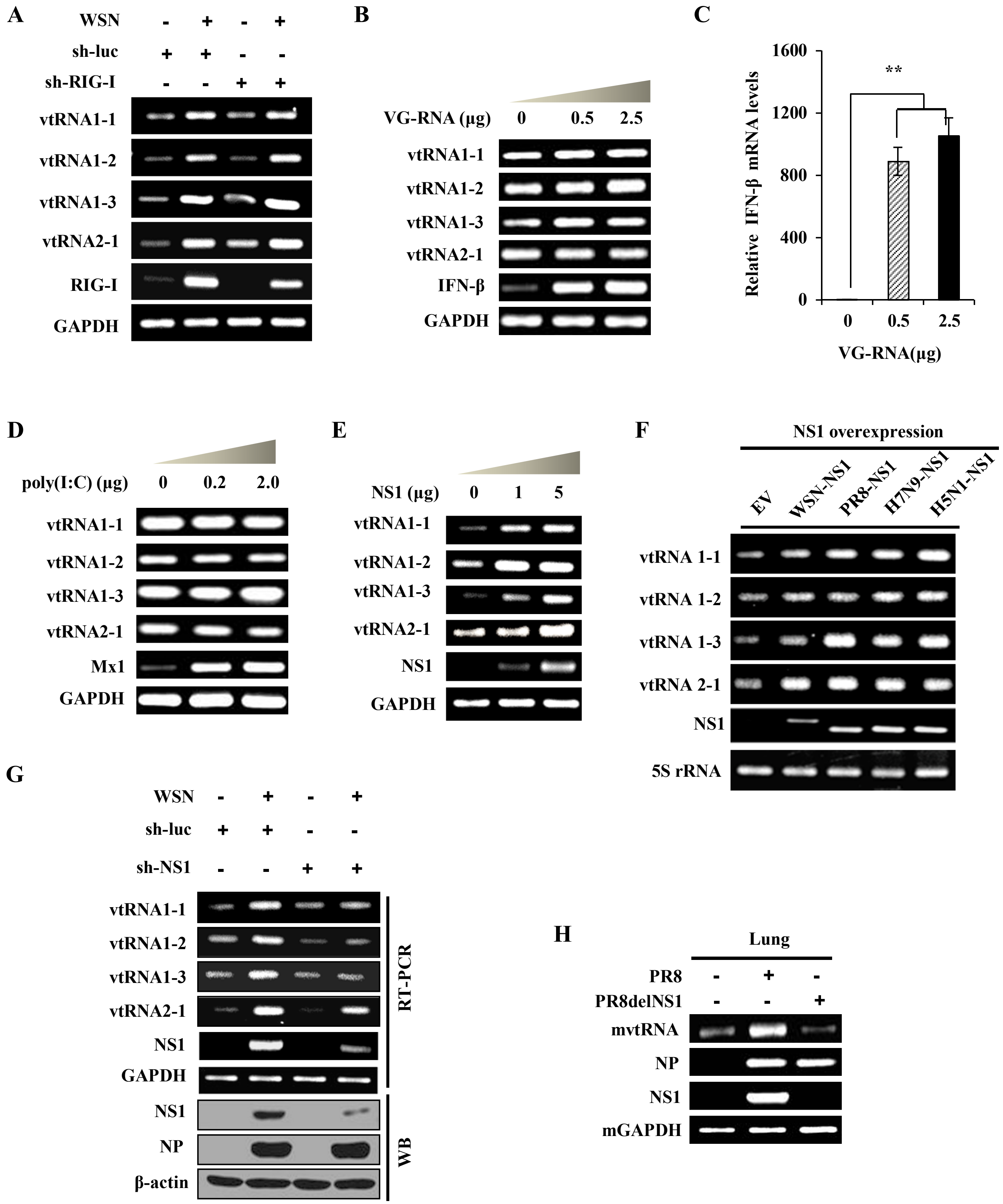
A
WSN - + - +
sh-luc + + - -
sh-RIG-I - - + +
vtRNA1-1
vtRNA1-2
vtRNA1-3
vtRNA2-1
RIG-I
GAPDH
B
VG-RNA (μg) 0 0.5 2.5
vtRNA1-1
vtRNA1-2
vtRNA1-3
vtRNA2-1
IFN-β
GAPDH
C
Relative IFN-β mRNA levels
1600
1200
800
400
0
**
0 0.5 2.5
VG-RNA(μg)
D
poly(I:C) (μg) 0 0.2 2.0
vtRNA1-1
vtRNA1-2
vtRNA1-3
vtRNA2-1
Mx1
GAPDH
E
NS1 (μg) 0 1 5
vtRNA1-1
vtRNA1-2
vtRNA1-3
vtRNA2-1
NS1
GAPDH
F
NS1 overexpression
EV
WSN-NS1
PR8-NS1
H7N9-NS1
H5N1-NS1
vtRNA 1-1
vtRNA 1-2
vtRNA 1-3
vtRNA 2-1
NS1
5S rRNA
G
WSN - + - +
sh-luc + + - -
sh-NS1 - - + +
vtRNA1-1
vtRNA1-2
vtRNA1-3
vtRNA2-1
NS1
GAPDH
RT-PCR
NS1
NP
β-actin
WB
H
Lung
PR8 - + -
PR8delNS1 - - +
mvtRNA
NP
NS1
mGAPDH

Figure S5

A

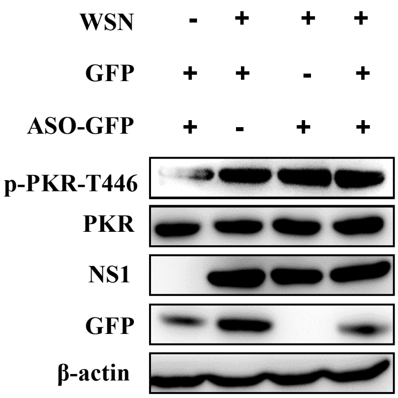

B

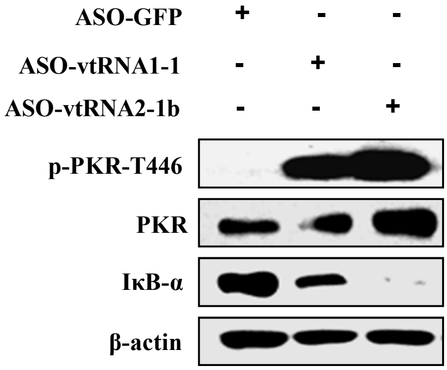

C

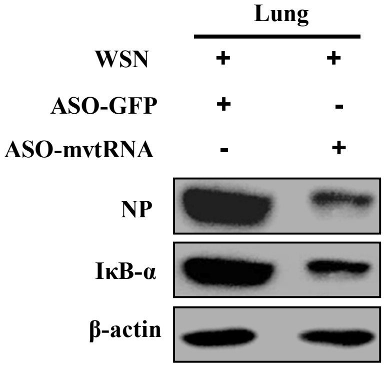

Figure S6

A

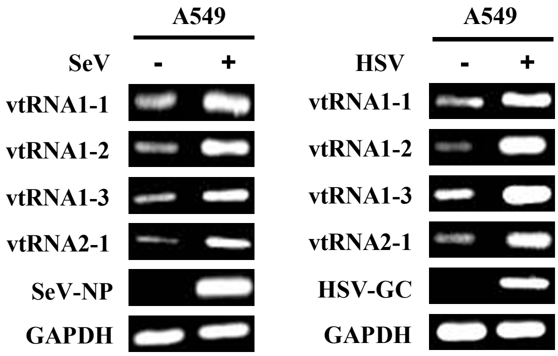
A549
SeV - +
vtRNA1-1
vtRNA1-2
vtRNA1-3
vtRNA2-1
SeV-NP
GAPDH
A549
HSV - +
vtRNA1-1
vtRNA1-2
vtRNA1-3
vtRNA2-1
HSV-GC
GAPDH

Figure S7

A

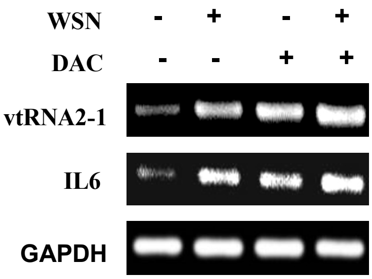

Figure S8

A

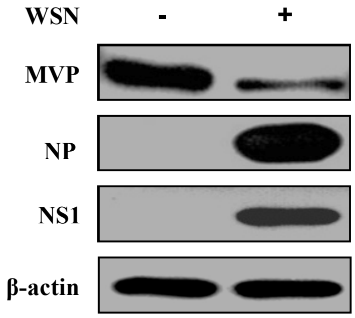

Supplement: SUPPLEMENTARY DATA [file supp_gkv1078_nar-01559-x-2015-File010.pdf]
